# Computing Influential Nodes Using the Nearest Neighborhood Trust Value and PageRank in Complex Networks

**DOI:** 10.3390/e24050704

**Published:** 2022-05-16

**Authors:** Koduru Hajarathaiah, Murali Krishna Enduri, Satish Anamalamudi, Tatireddy Subba Reddy, Srilatha Tokala

**Affiliations:** 1 Department of Computer Science and Engineering, SRM University-AP, Amaravati 522502, India; hazarathaiah_koduru@srmap.edu.in (K.H.); satish.a@srmap.edu.in (S.A.); srilatha_tokala@srmap.edu.in (S.T.); 2 Department of Computer Science and Engineering, B V Raju Institute of Technology, Medak 502313, India; subbareddynec@gmail.com

**Keywords:** trust value, PageRank, similarity ratio, centrality measure, influential nodes, complex networks

## Abstract

Computing influential nodes gets a lot of attention from many researchers for information spreading in complex networks. It has vast applications, such as viral marketing, social leader creation, rumor control, and opinion monitoring. The information-spreading ability of influential nodes is greater compared with other nodes in the network. Several researchers proposed centrality measures to compute the influential nodes in a complex network, such as degree, betweenness, closeness, semi-local centralities, and PageRank. These centrality methods are defined based on the local and/or global information of nodes in the network. However, due to their high time complexity, centrality measures based on the global information of nodes have become unsuitable for large-scale networks. Very few centrality measures exist that are based on the attributes between nodes and the structure of the network. We propose the nearest neighborhood trust PageRank (NTPR) based on the structural attributes of neighbors and nearest neighbors of nodes. We define the measure based on the degree ratio, the similarity between nodes, the trust values of neighbors, and the nearest neighbors. We computed the influential nodes in various real-world networks using the proposed centrality method. We found the maximum influence by using influential nodes with SIR and independent cascade methods. We also compare the maximum influence of our centrality measure with the existing basic centrality measures.

## 1. Introduction

Understanding the dynamics of information spreading in technological, biological, and social networks become one of the most important topics for large-scale networks [1,2,3]. Thus, we studied the dynamics of information spreading by using influential nodes. The information spreading ability of influential nodes is greater than that of other nodes in the network. In this context, computing influential nodes in any network is important to many researchers. We can also study or understand the attributes and characteristics of the network while computing the influential nodes in complex networks [4,5]. Several researchers proposed centrality measures to identify the influential nodes in a complex network. The most commonly used centrality measures are the degree centrality [6], closeness centrality [7], betweenness centrality [8], and semi-local centrality [9]. PageRank centrality [10] and leader-rank centrality [11] were proposed based on the importance of the quality and quantity of the node’s neighbors. PageRank is a measure of a page that is measuring the quality or quantity of that page [10,12]. Zhang et al. [13,14] defined a measure called H-index, which is an indicator of the citation pattern of the paper. Nomura et al. [15] defined a hyperlink induced topic search (HITS) method which is based on the hyperlink structures of web pages.

Various measures and methods are focused on the network’s structure, and very few measures are based on local information or local attributes of the node in the network [16,17]. Some of the commonly used centrality measures are defined based on local information, such as degree centrality, clustering coefficient [18,19], semi-local centrality, normalized local centrality [20], local neighbor contribution [21], and local centrality with coefficient [22]. The degree centrality measure has low accuracy due to consideration of first-order neighbors [18]. Due to the increased time complexity, centrality measures based on global information of nodes have become unsuitable for large-scale networks. In the current scenario, the challenging task is to find the influential nodes with high accuracy and in less running time. The *k*-core measure [23] also concentrates only on network structure. Various local centrality measures are listed in Table 1. There exist very few centrality measures that are based on the structure of the network and information between nodes [24,25,26].

In social networks, information is spread between two individual people based on similar behavior or other similarities [17,30,31]. Zhao et al. [16] gave a centrality measure based on structural similarity for finding influential nodes. The K–L divergence [32] is used to measure the structural similarity of nodes, and enhanced PageRank is used to rank the nodes in large-scale networks. Sheng et al. [28] defined the trust–PageRank (TPR) measure based on attribute information between nodes and network structure. Trust–PageRank creates a relation between the trust value and the PageRank of a node in the network. The trust value of two adjacent nodes is defined by the similarity ratio and the degrees of those nodes. The similarity ratio of two individual nodes is similar to the characteristics of these nodes. The construction of a measure of trust–PageRank mainly depends on the degree and similarity, which play a key role in spreading the information in the network. During the construction of a measure trust–PageRank, they considered only single a neighborhood level. Thus, we explore this measure not only at one neighborhood level but also the second neighborhood level. This idea can be further extended to other neighborhood levels too.

**Our Contribution:** In this research work, we propose a nearest neighborhood trust PageRank (NTPR) method which considers not only neighborhood trust values at the one-neighborhood level but also the nearest neighbors of trust values up to the second-neighborhood level. In this work, the degree ratio is also defined, along with next neighbor levels, instead of adjacent nodes. Considering the greater number of neighbors, the degree ratio and trust value capture information spread more accurately. We notice first-level neighbors and second-level neighbors’ information plays a crucial role in the influence of a node. Additionally, our method uses the trust value, which includes a degree ratio with neighbors, the similarity ratio, and the second-level neighbors’ information. We proposed an enhanced version of trust–PageRank, which is a nearest neighborhood trust PageRank measure to compute the influential nodes in complex networks [29]. By using the proposed centrality, we computed the influential nodes in various real-world networks. We found the maximum influence of influential nodes by using the SIR, independent cascade, and greedy methods. In [29], we defined the NTPR measure. In this paper, we provide results and comparisons with other basic centralities. We also compare our centrality measure with existing basic centrality measures, and it produces greater influence than the others which are discussed in the coming sections.

The rest of the paper is structured as follows: We list some of the existing basic centralities in Section 1.1. In Section 2, we define the nearest neighborhood trust page centrality measure and design an algorithm for computing the measure of each node in the network. We listed some of the network datasets which we used for our experiments and SIR, the independent cascade model, and Kendall’s tau details are given in Section 3. In Section 4, we discuss the correlations between NTPR and various basic centrality measures. We observe the difference between infection rate and centrality value with SIR and the independent cascade model. We also explain the performance of NTPR with various basic methods and the maximum influence levels of nodes at different infection rates. Finally, the conclusions are discussed, along with future research work.

### 1.1. Related Work

In this section, we list a few centrality methods and consider unweighted and undirected networks. Let G=(V,E) be a network, where *V* denotes the vertices and *E* denotes the edges of network *G*. The degree centrality (DC) [6] denotes the number of nodes adjacent or directly connected to a node. The closeness centrality (CC) [7] of a node is how closely it is connected to other nodes using distance which is computed within the graph. The closeness centrality (CC) of node *v* defined as

$$CC\left(v\right)=\frac{1}{\sum_{v_{i}\in V}d(v,v_{i})}$$

where $d(v,v_{i})$ denotes the distance between vertices $v_{i}$ and *v*. The betweenness centrality (BC) [8] of the vertex is a measure of the ratio of the shortest path involving the vertex to all the shortest paths between every pair of vertices. The betweenness centrality (BC) of node *v* can be calculated as

$$BC\left(v\right)=\sum_{v_{i}\neq v_{j}\neq vs.\in V}\frac{d_{v_{i}v_{j}}\left(v\right)}{d_{v_{i}v_{j}}}$$

where $d_{v_{i}v_{j}}$ is the shortest path from the vertex $v_{i}$ to $v_{j}$ (or $v_{j}$ to $v_{i}$ ), and $d_{v_{i}v_{j}}\left(v\right)$ is the shortest path between vertices $v_{i}$ and $v_{j}$ passing through vertex *v*. The semi-local centrality (SC) [9] is defined by the number of neighbors up to two levels, and vertex *v* is computed as

$$SC\left(v\right)=\sum_{v_{i}\in N_{v}}\sum_{v_{j}\in N_{v_{i}}}NN\left(v_{j}\right)$$

where $N_{v}$ and $N_{v_{i}}$ are sets of adjacent nodes to vertices *v* and $v_{i}$, respectively; and $NN(v_{j})$ represents the second-level neighbors of vertex $v_{j}$. PageRank measures the quality or quantity of a page [10,12]. PageRank is used for webpage sorting and for ranking data on various networks’ webpages.

$$PR_{v}^{t}=\frac{1-\alpha }{n}+\alpha \sum_{v_{i}\in N_{v}}\frac{PR_{v_{i}}^{t-1}}{k_{v_{i}}}$$

where *n* is number of vertices in a network, $N_{v}$ represents the neighbors of vertex *v*, α is the jump probability, $k_{v_{i}}$ is the number of vertices to which the vertex $v_{i}$ points, and *t* represents an iterative parameter. The first term in PageRank is for regularizing the PageRank, and the sum should become one when it is maximized.

The trust–PageRank [28] method is defined based on involving the trust value in the PageRank method. The intuition behind including the trust value is that if the vertex has more trust value, it receives more information from the other vertices. This is due to information spreading through the neighbor nodes within the network. The trust–PageRank [28] method is defined as follows:

(1) $$TPR_{v}^{t}=\frac{1-\alpha }{n}+\alpha \sum_{v_{i}\in N_{v}}T\left(v,v_{i}\right)TPR_{v_{i}}^{t-1}$$

where $T(v,v_{i})$ denotes the trust values of nodes *v* and $v_{i}$, *n* is the number of nodes, $N_{v}$ is set of adjacent nodes of *v*, α denotes jump probability, and *t* represents an iterative parameter. The first term in TPR is for regularizing the TPR, and the sum should be one when it is maximized.

## 2. A Centrality Measure Using Second-Level Neighborhood Trust Values

We define a nearest neighborhood trust PageRank (NTPR) method which is constructed using the trust values of neighbors up to the second-level. The degree ratio and similarity ratio of neighbors of the node up to the second-level play a crucial role in constructing the centrality measure NTPR. The trust values of neighbors mainly depend on the degree ratio and similarity ratio. In the article [28], the trust–PageRank (TPR) method was defined. In this research, other levels of adjacent neighborhood attributes were missing. Our intuition is that if we insist on second-level neighborhood trust values, then we could observe the influence maximization by computing the seed nodes or influential nodes in the complex network. We can also apply this logic to getting further level neighborhood information on trust values. However, time complexity may increase if we increase the number of levels.

**Similarity Ratio:** The similarity ratio of a vertex $v_{i}$ and an adjacent neighbor $v_{j}$ is the similarity of $v_{i}$ and $v_{j}$ divided by the addition of the similarity between the adjacent neighboring vertex $v_{j}$ and its adjacent neighbor vertices $v_{l}$. The similarity ratio can be measured as follows:

(2) $$SR\left(v_{i},v_{j}\right)=\frac{S(v_{i},v_{j})}{\sum_{v_{l}\in N_{v_{j}}}S\left(v_{j},v_{l}\right)}$$

The SimRank [33] is constructed to measure the similarity of two nodes. In the SimRank method, if any two nodes are similar, then both are related to each other with some common attribute information or characteristics. The similarity between $v_{i}$ and $v_{j}$ can be calculated as:

(3) $$S\left(v_{i},v_{j}\right)=\left\{\begin{array}{cc} \frac{1}{|N_{v_{i}}\left|\right|N_{v_{j}}|}\sum_{v_{l}\in N_{v_{i}}}\sum_{v_{m}\in N_{v_{j}}}S\left(v_{l},v_{m}\right), & \mathrm{if}\;v_{i}\neq v_{j}\\ 1, & \mathrm{if}\;v_{i}=v_{j} \end{array}\right.$$

where $N_{v_{i}}$ and $N_{v_{j}}$ sets of adjacent vertices of $v_{i}$ and $v_{j}$, respectively; $S(v_{l},v_{m})$ is the similarity of $v_{l}$ and $v_{m}$. In Equation (3), computing $S(v_{i},v_{j})$ is an iterative process. In this iteration, we consider $S(v_{i},v_{j})$ is 1 if $v_{i}=v_{j}$ and $S(v_{i},v_{j})$ is 0.1 if $v_{i}\neq v_{j}$.

**Degree ratio:** The degree ratio of a vertex $v_{i}$ and an adjacent vertex $v_{j}$ is the ratio of the degree of vertex $v_{i}$ to the sum of the degrees of adjacent vertices of $v_{j}$. For normalization of this degree ratio, we use the sum of the degrees of the neighbors of $v_{j}$ and the sum of the degrees of the second-level neighbors of $v_{j}$. The degree ratio can be defined as:

(4) $$DR\left(v_{i},v_{j}\right)=\frac{\sum_{v_{l}\in N_{v_{i}}}d_{v_{l}}}{\sum_{v_{m}\in N_{v_{i}}}d_{v_{m}}+\sum_{v_{m}\in N_{v_{j}}}\sum_{v_{n}\in N_{v_{m}}}d_{v_{n}}}$$

**Trust value:** Trust values of vertices $v_{i}$ and $v_{j}$ are defined by the similarity ratios and degree ratios of $v_{i}$ to $v_{j}$. Trust value is calculated as follows:    

(5) $$TV\left(v_{i},v_{j}\right)=\left(1-k\right)SR\left(v_{i},v_{j}\right)+kDR\left(v_{i},v_{j}\right)$$

where $SR(v_{i},v_{j})$ and $DR(v_{i},v_{j})$ are degree ratios and similarity ratios of vertices $v_{i}$ and $v_{j}$, respectively. The parameter *k* value is in between 0 and 1 and this value is taken to be k=0.85. Now, we propose nearest neighborhood trust–PageRank (NTPR) using trust value of adjacent vertices up to the second-level, as follows:

(6) $$\begin{array}{cc} NTPR_{t}\left(v_{i}\right)= & \frac{1-\alpha }{\left|V\right|}+\frac{\alpha }{{\left|V\right|}^{2}}[\sum_{v_{j}\in N_{v_{i}}}TV\left(v_{i},v_{j}\right)NTPR_{t-1}\left(v_{j}\right)\\ & +\sum_{v_{j}\in N_{v_{i}}}\sum_{v_{l}\in N_{v_{j}}}TV\left(v_{l},v_{j}\right)NTPR_{t-1}\left(v_{l}\right)] \end{array}$$

where $TV(v_{i},v_{j})$ represents the trust values of nodes *v* and $v_{j}$; |V| represents the number of vertices; α indicates jump probability, and we consider this value to be 0.85. The first term in NTPR is a regularizing term, the second term of NTPR contains neighbors’ similarity and degree ratios, and the third term of NTPR contains second-level neighbors’ similarity and degree ratios. Our intuition to define Equation (6) is if we insist on second-level neighborhood information of trust value, then we can observe the influence maximization by computing the seed nodes or influential nodes in the complex network. We could also extended it up to further levels of neighborhood information of trust value. In Equation (6), computing $NTPR_{t}$ is in iterative relation, and we consider the number of iterations *t* from 0 to 1000. We can consider beyond 1000, but we considered up to 1000 to simplify our simulations. We initialized the $NTPR_{0}$ at every vertex to 0.1. For more details, see Algorithm 1.
**Algorithm 1:** Computing $NTPR_{t}$ for every vertex of graph *G***Input**: Graph G=(V,E) with vertices and edges**Output**: $NTPR_{t}$ for every vertex of graph *G*
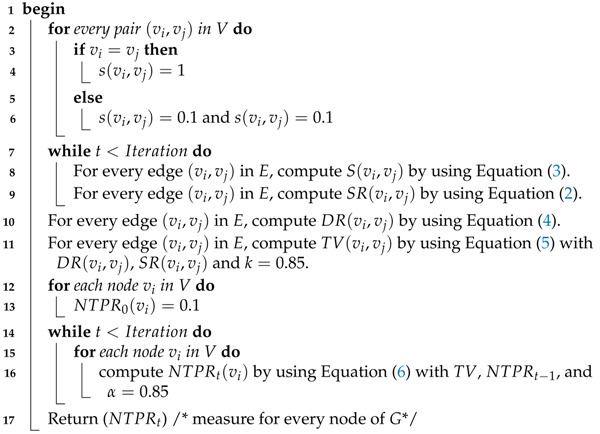


**Figure 1:**
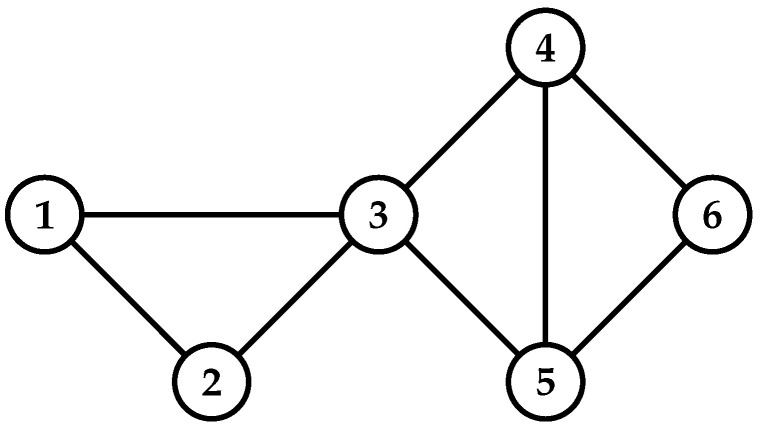
A graph with 6 vertices and 8 edges.

**Figure 2:**
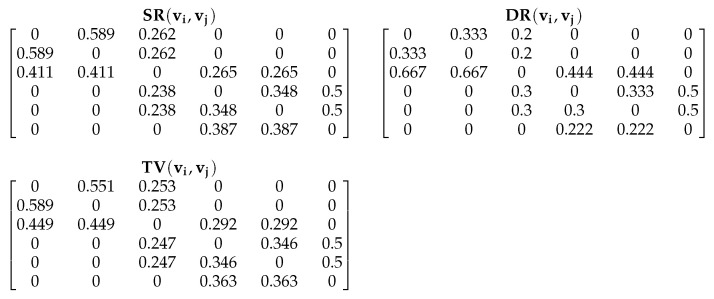
Degree ratio $DR(v_{i},v_{j})$, similarity ratio $SR(v_{i},v_{j})$, and trust value $TV(v_{i},v_{j})$ for every pair of nodes of the graph in Figure 1.

Algorithm 1 is the procedure for computing the NTPR measure for each vertex in the network. We illustrate Algorithm 1 by using an example that is given in Figure 1. Finding NTPR is depends on trust values. Each trust value is based on similarity and degree ratio. For Figure 1, we used Equations (2) and (4) to calculate the similarity ratio $SR(v_{i},v_{j})$ and degree ratio $DR(v_{i},v_{j})$ for every pair of vertices. We found trust values $TV(v_{i},v_{j})$ by using Equation (5) with the help of $DR(v_{i},v_{j})$, $SR(v_{i},v_{j})$, and k=0.85 for every pair of vertices in the network, as shown in Figure 2. We found the NTPR measure for every vertex using Equation (6). For the network in Figure 1, NTPR values for all nodes are as follows: $\left\{0.22398,0.22398,0.40797,0.33179,0.33179,0.22757\right\}\times 10^{-23}$. Thus, vertex 3 has the highest NTPR value, i.e., $0.40797\times 10^{-23}$, and the sequence of influenced nodes of the network in Figure 1 is {3,4,5,6,1,2}. In our simulations, the number of iterations was set to 1000. The combination depends on the value of the damping factor, which is between 0 and 1. The damping factor α [34] specifies how long a random web surfer spends following the hyperlink structure rather than teleporting. If we consider the damping factor to be 0.8, that means out of total time, 80% of the time has been taken by a random web surfer to follow the hyperlink structure, and the remaining 20% of the time they teleport to new web pages randomly. To maximize the influence by computing the influential nodes in the complex network and to avoid the various parameters in our simulations, we fixed the values of *k* and α to 0.85 [34]. Complexity will increase if we tune all these parameters.

### Time Complexity of NTPR

Consider a graph G=(V,E) with |V|=n and the maximum degree of *d*. Clearly, we can observe that the time complexities of measures SR, DR, and TV are $O(d^{2})$, $O(d^{3})$, and $O(d^{3})$, respectively. Thus, the time complexity to find the $NTPR_{t}$ for a vertex is $O(td^{5})$. To find the $NTPR_{t}$ measure for any vertex in the graph *G* is $O(td^{5}n)$. We considered *t* constant in our algorithm, and the time complexity of Algorithm 1 is $O(d^{5}n)$, where *d* is maximum degree and *n* is the number of vertices of the graph.

## 3. Details on Implementation

Description of Datasets: To test our suggested centrality NTPR, we used four datasets [35] in a performance evaluation. The four datasets were email-univ, euroroad, powergrid, and web-polblogs which are provided at https://networkrepository.com/ on 19 March 2020. Table 2 summarizes the details of the networks.

**Susceptible–Infected–Recovered Model:** In this work, we investigated the dynamics of information spreading by using the SIR simulation model [36,37]. The SIR model is commonly used for how much information is spread with in the network. This model is used to understand the dynamics of the spreading of diseases and to find a total number of infected nodes at different infection probabilities. The SIR model is divided into 3 components, susceptible, infected, and recovered. The susceptible part tells us that no infection has taken place. The term “infected” refers to infections spread over the network by others. Finally, recovered means the cured individuals, and they do not infect after a certain number of rounds. At the starting stage, seed nodes will be infected, which can help us to find the spreading capability. These infected nodes later in each iteration infect their neighbors with a certain probability in the network. The number of infected nodes grows over time until it reaches a stable state. The SIR model is one of the methods used to estimate centrality measurements in terms of network performance.

**Independent Cascade Model (IC):** The IC model is a stochastic technique in which data are transferred from one node to another depending on probabilistic criteria [3]. Li et al. [38] categorized the diffusion models into two types, predictive and explanatory models. The IC model is a predictive model that uses specific parameters to estimate the forecast information diffusion process and influence maximization in social networks. The IC model’s information diffusion operates as follows: The network’s nodes can be in one of two states: active or inactive. If a node accepts the information being circulated in the network, it is deemed active; if it does not have information, it is considered inactive. Thus, an independent cascade model, which is a form of an epidemic model, proposes that an individual will achieve innovation with a specific probability if at least one of its neighbors has done so. We compared this model with the SIR model.

**Greedy Model:** The greedy algorithm implements the problem-solving strategy of choosing the locally best option at every stage [3]. A greedy strategy may not generate an optimal result in many cases, but it can produce locally optimized solutions that resemble globally optimal solutions in acceptable time frames. Greedy algorithms are usually less computationally efficient than other techniques, such as dynamic programming, but they often compromise the quality of the solution to achieve speed. This algorithm was used to find top-ten seed nodes. The greedy model evaluates the incremental spread of each node separately rather than as a whole. It determines the maximum information spread for all remaining candidate nodes before selecting the node with the highest spread. The calculation of the spread for all nodes uses iterations and selects the top 10 nodes in terms fo influence. We compare the maximum influence with the greedy approach with that found by our centrality measure.

**Kendall’s tau (τ):** This is used to determine how closely two ranking lists rate the same set of items [39,40]. Kendall’s tau (τ) measures how many concordant and discordant ranking pairs there are in each of the two lists. Kendall’s tau (τ) [41] is defined as:

$$\begin{array}{c} \tau =\frac{\sum_{i<j}[\mathrm{sgn}\left[\left(P_{i}-P_{j}\right)\left(Q_{i}-Q_{j}\right)\right]}{0.5(N(N-1))} \end{array}$$

where sgn(P) is a sign function, if P>0 returns 1, if P<0 returns −1, and if P=0 returns 0. $P_{i}$ and $P_{j}$ are the ranks of nodes *i* and *j* in ranking list one. $Q_{i}$ and $Q_{j}$ are the ranks of nodes *i* and *j* in ranking list two, and *N* is the number of nodes. Let us take two variables, *P* and *Q*. *Case 1:* if $\left(P_{i}-P_{j}\right)\left(Q_{i}-Q_{j}\right)>0$, both nodes are concordant pairs in ranking lists one and two. *Case 2:* if $\left(P_{i}-P_{j}\right)\left(Q_{i}-Q_{j}\right)<0$, both nodes are discordant pairs in ranking lists one and two. *Case 3:* if $\left(P_{i}-P_{j}\right)\left(Q_{i}-Q_{j}\right)=0$, both nodes are in the same rank in ranking lists one and two.

## 4. Results

In this section, we show the results on the correlations of our method NTPR with other different existing centrality measures. We show that the spread of information increases with the value of the centrality of a node in the network. Based on the SIR simulation and independent cascade models, we compared cumulative infected nodes for NTPR, BC, CC, DC, SC, PR, and TPR centralities, and the greedy approach. We studied the pattern of maximum influence with different infection rates for these centralities.

### 4.1. Correlations of the NTPR Method with Various Centrality Measures

**Figure 3:**
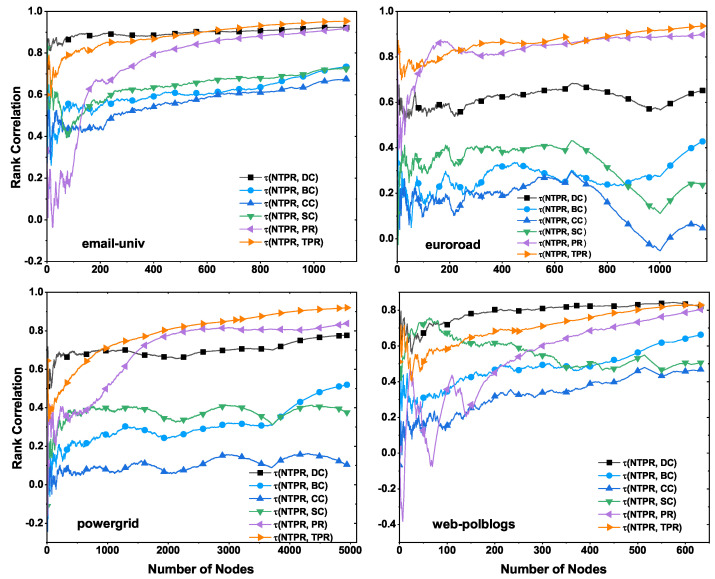
Correlation (τ) between NTPR with DC, BC, CC, SC, PR, and TPR and top nodes.

We show the results of correlations between the NTPR method and other basic centrality methods, such as TPR, PR, BC, DC, CC, and SC, on various real-world networks, in Table 3 and Figure 3. We computed the ranking of each vertex by using these centralities in the network. In Figure 3, we show correlation plots of the NTPR method and basic centrality methods for every top-*N* vertices, where N={1,2,⋯,n} and *n* is the number of nodes in the network. For every vertex, the correlation value of the NTPR method and each basic centrality method is shown in Table 3. In Table 3 and Figure 3, we can observe the close connection between any basic centrality method and the NTPR method. In Figure 3 and Table 3, we see that the NTPR measure is similar to PR for email-univ, euroroad, and powergrid datasets. We can also observe that NTPR is similar to DC for email-univ and web-polblogs datasets. This is because NTPR is an improved version of PageRank with degree centrality. The NTPR method does not have a close correlation with any of the other centralities. Degree centrality is primarily correlated with the NTPR measure at the initial set of vertices in the powergrid network and email-univ. Degree centrality is highly correlated with the NTPR method in the second half of set of vertices in the web-polblogs network. Thus, the NTPR measure does not closely correlate with existing basic centralities except PR and DC.

**Figure 4:**
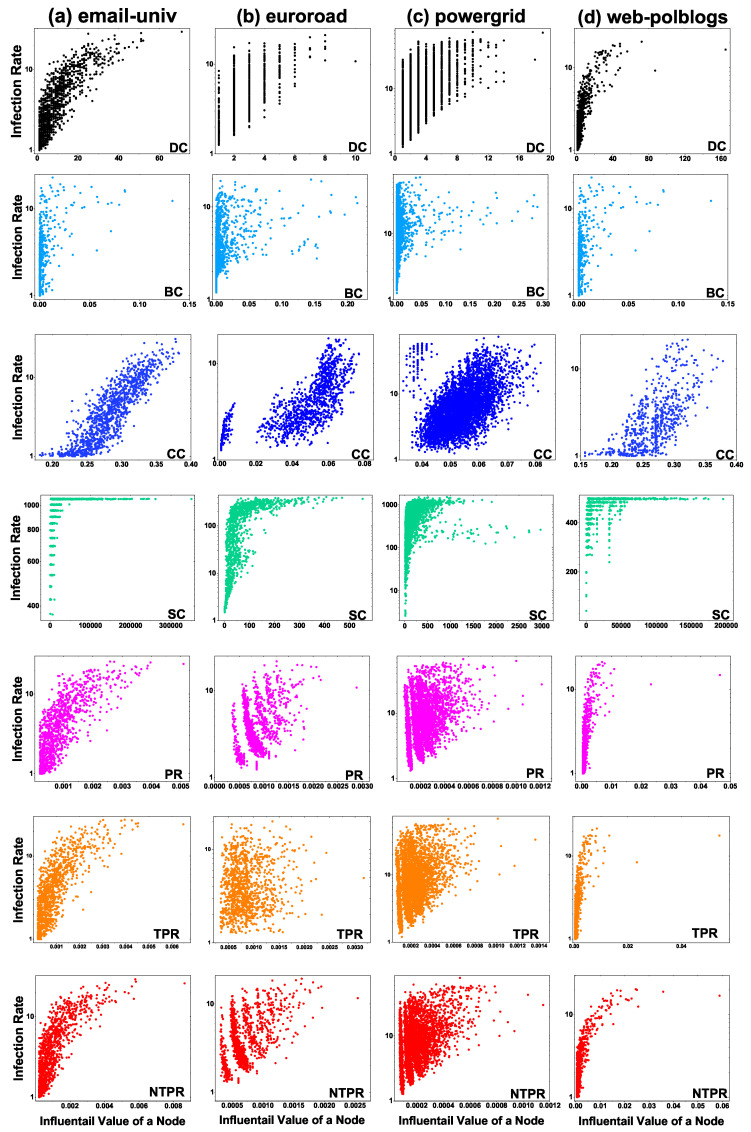
Centrality value with infection rate according to SIR simulations in four networks, column-wise.

We show that the information-spreading rate of the NTPR method is higher than those of the BC, DC, SC, CC, PR, and TPR methods with the help of SIR simulations. We computed the centrality value for each node by using each centrality method. The infected node was the single node with the highest centrality value. The cumulative infection was calculated by running SIR simulations 100 times. We took infection probability β to be in between 0.1 and 0.4. Figure 4 shows a plot of the centrality value of nodes with increasing infection rate. Our method NTPR, TPR, PR, and DC measures had good connections compared with SC, BC, and CC in the email-univ network, as shown in Figure 4a. The NTPR method shows more information spreading when we compare it with TPR, PR, SC, CC, BC, and DC in the euroroad network (see column b in Figure 4). We can observe that, if the node has a high NTPR centrality value, then the node has a high information propagation capability (see Figure 4c). The centralities DC and BC have poor information spreading capabilities when compared with other centralities in the powergrid network, which can be observed in Figure 4c. The CC, DC, TRP, PR, and NTPR measures show good cumulative infection compared to SC and BC methods in web-polblogs. For the web-polbogs network, SIR simulation plots are given in Figure 4d. The NTPR centrality method provides more information spreading in the euroroad and powergrid networks. Clearly, we can observe that information spreading increases with the node centrality measure NTPR in four different real-world networks.

### 4.2. Cumulative Infected Nodes for the Proposed Centrality and Basic Centralities

**Figure 5:**
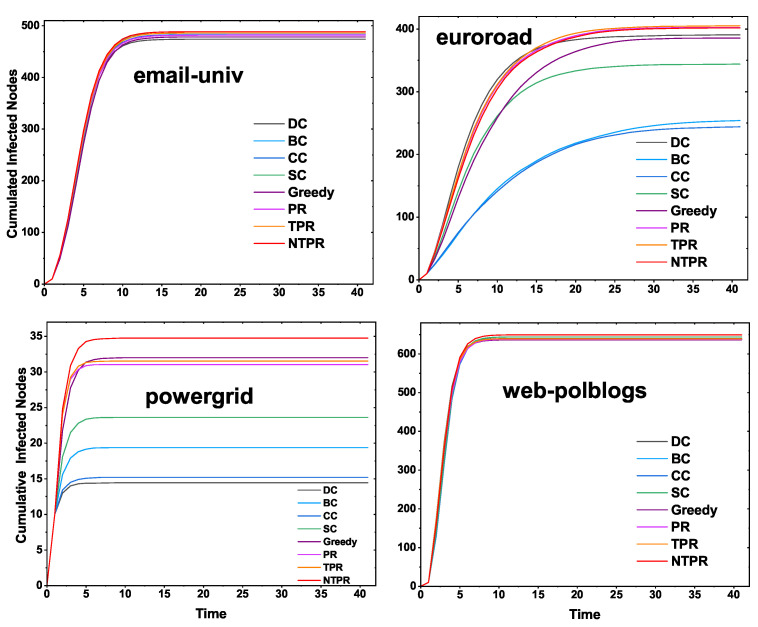
Cumulative infected nodes of the SIR model according to NTPR and other centralities for four real-world networks (top-10 seed nodes).

In this sub-section, we show the cumulative infected nodes or the effect of spreading the information while initially infected by the top-ten seed node or influential nodes. We computed the top-ten most influential nodes or seed nodes by using the proposed centrality method (NTPR); basic centrality measures BC, CC, SC, DC, PR, and TPR; and a greedy method. With the help of the SIR model, initially, these top-ten seed nodes were infected. In the next time step, adjacent vertices of these seed nodes get infected with infection probability β. We consider infection probability β to be in between 0.1 and 0.4. After some time, whoever gets infected can recover with a certain recovery rate γ, which we have considered as one. The time steps were limited to 40, and we ran 100 simulations and found the average cumulative infected nodes. The results for four networks are displayed in Figure 5. The proposed centrality NTPR gives more cumulative infected nodes than TRP, PR, SC, CC, BC, and DC in euroroad and powergrid networks. The NTPR gives better results than the greedy method in the powergrid and euroroad networks. For email-univ and web-polblogs networks, our method provides similar results to the existing centralities. In email-univ and web-polblogs networks, the NTPR and greedy model give good results. The four networks’ results are displayed in Figure 5. In Figure 5, in most cases, information spreading with the proposed NTPR centrality method is dominating compared with the basic centrality measures.

### 4.3. Results on Spreading Information Rate vs. Centrality Value of a Vertex

**Figure 6:**
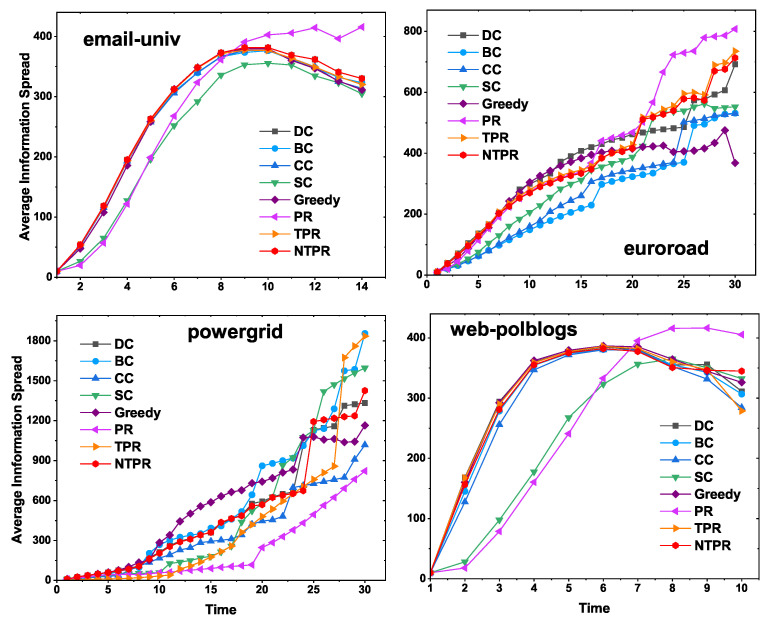
Average information spread among NTPR with other centralities for the four networks (top-10 seed nodes) using the independent cascade model.

In Figure 6, we show the average number of nodes at which information is received with different time steps by using the independent cascade model. The seed nodes were computed from different centrality measures which were input to the independent cascade model. In the independent cascade model, the number of iterations used for simulations was 1000. In email-univ and web-polblogs networks, the average information spread was greater at the initial steps for our centrality measure, and in the latter half, PR centrality dominated. However, NTPR and the greedy method both produced good results. The greedy method produced better results than basic centralities (DC, BC, CC, SC). In euroroad and powergrid, early on, our centrality measure produced good information spread, and in the second half of the time, greedy and PR performed well. In some cases, NTPR produced better average information spread than the other basic centrality measures with the independent cascade model. These results are shown in Figure 6.

**Figure A1:**
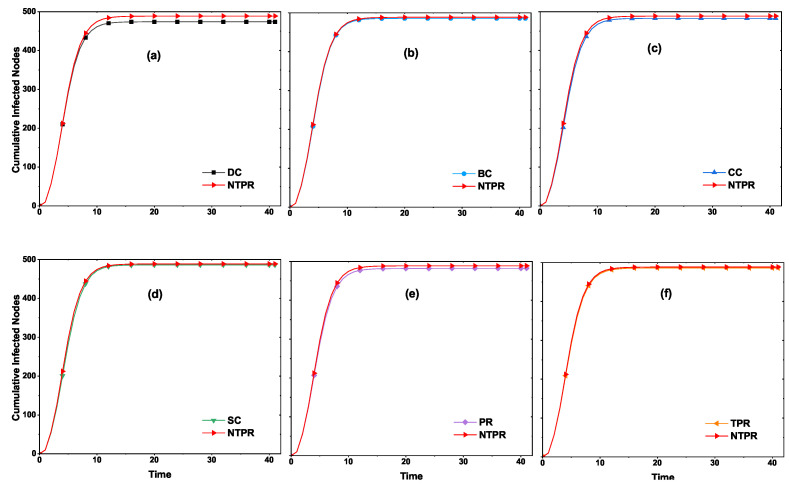
Cumulative infected nodes of the SIR model according to NTPR and other centralities for email-univ network (top-10 seed nodes).

**Figure A2:**
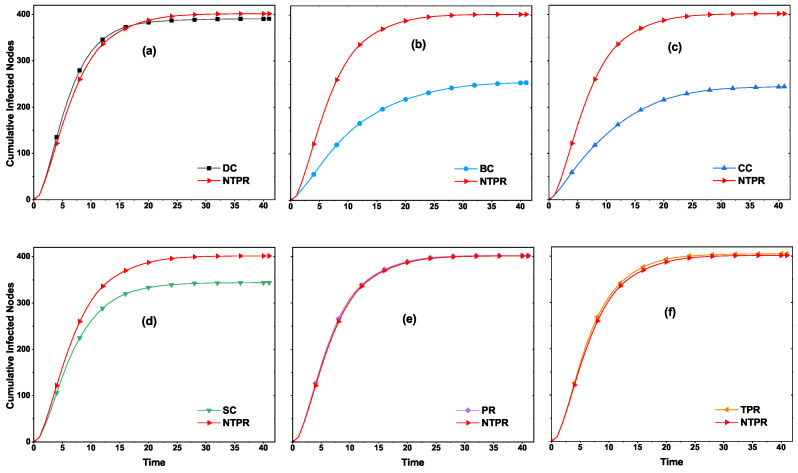
Cumulative infected nodes of the SIR model according to NTPR and other centralities for euroroad network (top-10 seed nodes).

**Figure A3:**
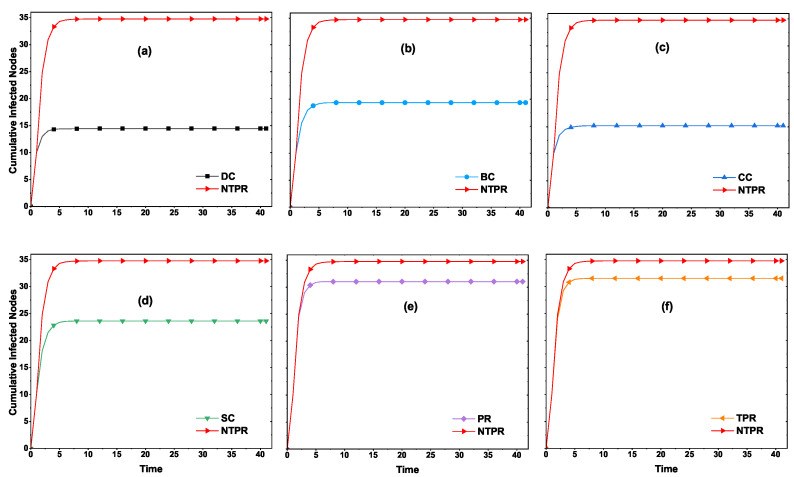
Cumulative infected nodes of the SIR model according to NTPR and other centralities for powergrid network (top-10 seed nodes).

**Figure A4:**
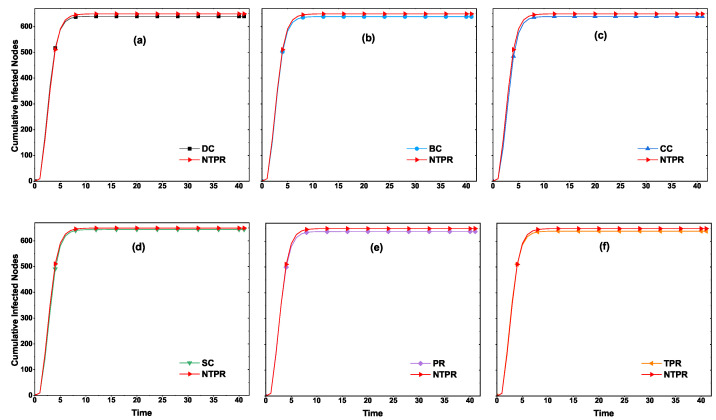
Cumulative infected nodes of the SIR model according to NTPR and other centralities for web-polblogs network (top-10 seed nodes).

**Figure A5:**
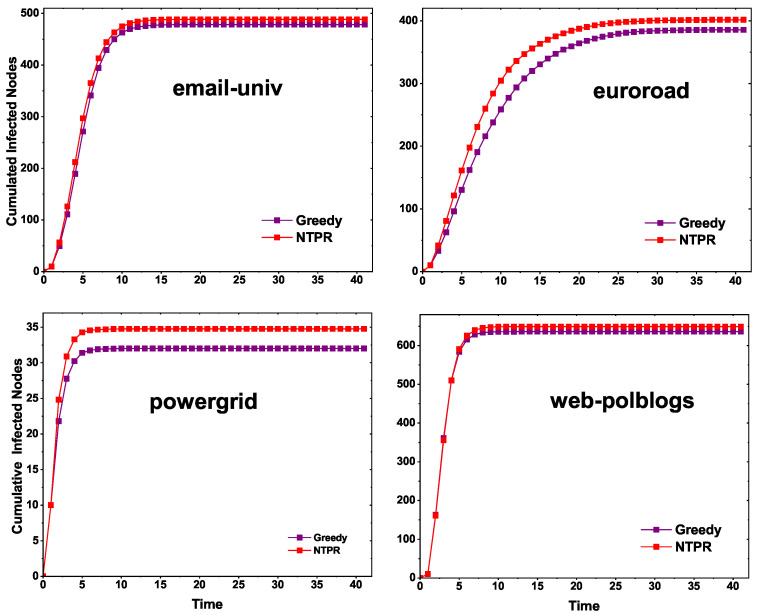
The top-10 seed nodes are initial infected nodes, identified by NTPR and a greedy algorithm using the SIR model for four datasets.

To more clearly understand, we have plotted for NTPR with basic centralities separately in Appendix A (see Figure A1, Figure A2, Figure A3, Figure A4 and Figure A5).

### 4.4. Maximum Influence at Various Infection Probabilities with Various Centrality Methods

The results of the evaluation of the top-10 most influential nodes of infection spreading ability are displayed in this section. The top-10 most influential nodes were discovered using several centralities, such as TPR, PR, DC, SC, CC, BC, and NTPR. From the information in the networks, we know that most influential nodes have the power to propagate. The SIR model was utilized, and the infection probability was considered to be between 0.1 and 0.4. We ran the SIR simulations with 100 iterations. To observe the effects of basic centralities and our new centrality, we found the maximum infection population at various infection probability levels. In this section, we talk about maximum information spread with different infection probabilities using the IC model. In this model, the infection probability is considered between 0.1 and 0.5. We ran the IC model simulations with 1000 iterations.

**Figure 7:**
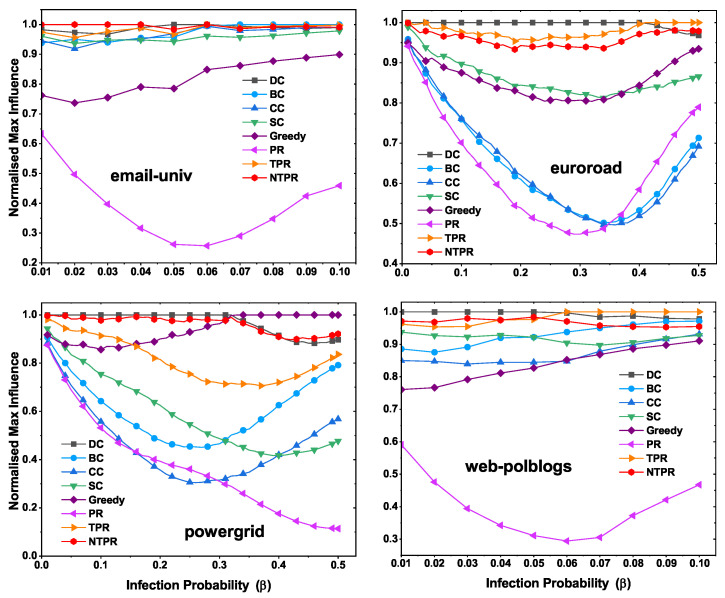
Normalized maximum influence levels of the top-ten most influential nodes of networks with various infection probabilities using the SIR model.

In Figure 7, we illustrate the normalized maximum infection with various infection probabilities. On the dataset email-univ, our measure NTPR produced good results compared to PR, TPR, SC, CC, BC, and DC, as shown in Figure 7. For the euroroad and web-polblogs datasets, DC performed well. However, our measure is close to DC centrality. On the powergrid network, NTPR took the top position over other centralities.

**Figure 8:**
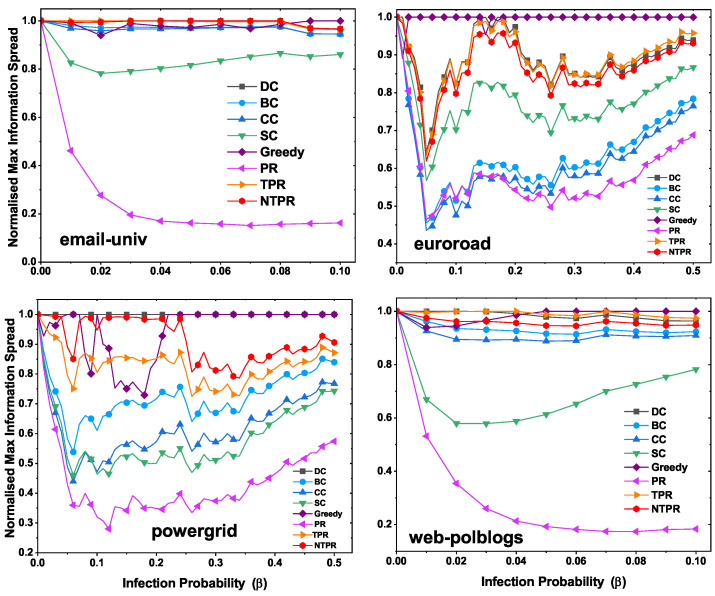
Normalized maximum information spread of the top-ten most influential nodes of networks with various infection probabilities using the independent cascade model.

In Figure 8, we illustrate the maximum information spread with various infection probabilities using the independent cascade model. In the email-univ network, the greedy and NTPR have more influence compared to other centralities. The greedy method has maximum information spread in euroroad and powergrid networks, which is shown in Figure 8. In webpolblogs networks, the greedy method is at the top position, and NTPR is close to the greedy method.

**Figure A6:**
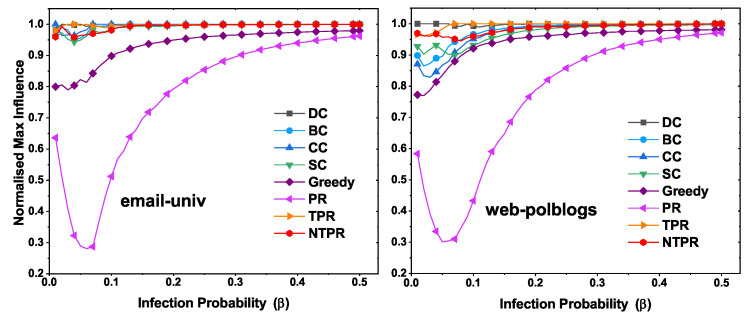
Normalized maximum influence levels of the top-ten most influential nodes of networks with various infection probabilities using the SIR model (email-univ and web-polblogs).

**Figure A7:**
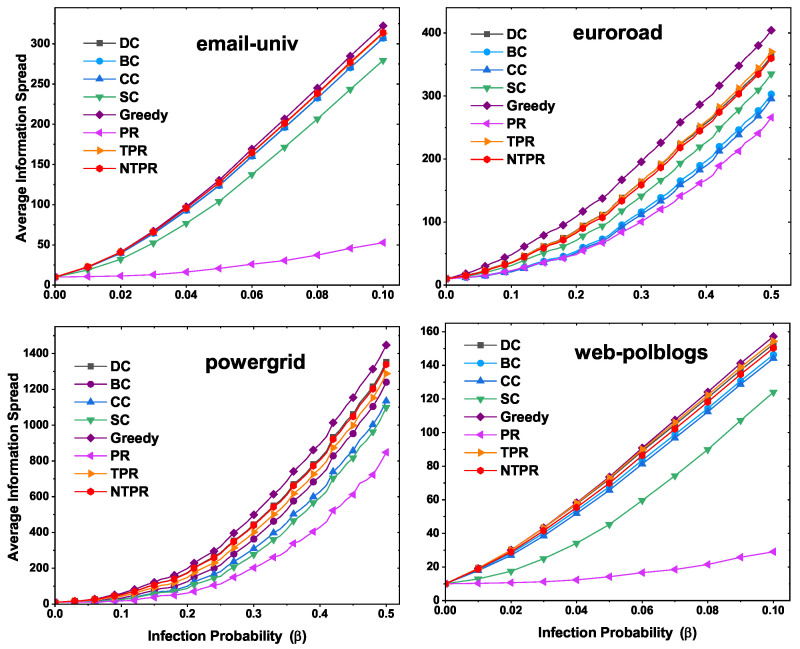
Average information spread of the top-ten most influential nodes of networks with various infection probabilities using the independent cascade model.

For clearer understanding, we show some of the figures for the two networks, email-univ and web-polblogs, in Appendix A (Figure A6); and for maximum information spread with various infection probabilities using the independent cascade model, we display figures for the four networks in Appendix A (Figure A7).

## 5. Conclusions

We focused on creating a centrality metric based on node attribute information and network topology. The degree ratio is utilized for node attribute information, whereas the similarity ratio is used for network structure information. The TPR was combined with trust value and PageRank in the previous work. However, we have focused on the importance of trust value in the nearest neighborhood throughout our work. The major goal of our metric is to evaluate trust value and PageRank, along with the closest neighbors. We use the degree ratio for second-level network neighbors as well. We can analyze the dynamics of spread inside the network by stepping up to the second-level. To assess performance, the SIR model and independent cascade model were used. Kendall’s tau was used to determine whether the NTPR and other existing basic centralities are similar. We obtained the experimental results by employing our nearest neighborhood trust metric, which is intended to identify the network’s most influential nodes.

Instead of using degree ratio, we would like to use closeness, betweenness, or other centrality measures to examine information spread with the proposed measure in the future. The value of the nearest neighborhood trust will also have the ability to expand to more layers of neighbors based on the diameter of the network or the maximum number of vertices of the network.

## Figures and Tables

**Table 1:** Different local centralities.

Local Centrality	Author and Year
Degree	Freeman et al., 1978 [6]
Semi-Local	Chen et al., 2012 [9]
Local Centrality with Coefficient	Zaho et al., 2017 [22]
Clustering Coefficient	Beralmand et al., 2018 [18,19]
Normalized Local Centrality	Zhao et al., 2018 [20]
Local Neighbor Contribution	Dai et al., 2019 [21]
PageRank	Xing et al., 2004 [27]
Trust–PageRank	Sheng et al., 2020 [28]
Nearest Neighborhood Trust Value	Proposed in this paper and Hajarathaiah et al., 2021 [29]

**Table 2:** Fundamental properties of four real networks.

Real Networks	Vertices	Edges	Maximum Degree	Average Degree	Avg. Cluster Coeff.
email-univ	1133	5451	71	9	0.220
euroroad	1174	1417	10	2	0.017
powergrid	4941	6594	19	2	0.080
web-polblogs	643	2280	165	7.09	0.232

**Table 3:** Correlation $\tau_{\left(\mathrm{NTPR},X\right)}$, where *X* is TPR, PR, SC, CC, BC, or DC centrality.

$\tau_{(\mathrm{NTPR},X)/}$ Networks	$\tau_{\left(\mathrm{NTPR},\mathrm{DC}\right)}$	$\tau_{\left(\mathrm{NTPR},\mathrm{BC}\right)}$	$\tau_{\left(\mathrm{NTPR},\mathrm{CC}\right)}$	$\tau_{\left(\mathrm{NTPR},\mathrm{SC}\right)}$	$\tau_{\left(\mathrm{NTPR},\mathrm{PR}\right)}$	$\tau_{\left(\mathrm{NTPR},\mathrm{TRP}\right)}$
email-univ	0.92	0.73	0.67	0.73	0.92	0.95
euroroad	0.65	0.43	0.05	0.24	0.89	0.94
powergrid	0.78	0.52	0.12	0.38	0.84	0.92
web-polblogs	0.82	0.66	0.47	0.51	0.81	0.83

## Data Availability

Not applicable.

## Abbreviations

Abbreviations used in this paper are as follows:

NTPR	Nearest Neighborhood Trust Page Rank
TPR	Trust–PageRank
SIR	Susceptible Infected Recovered
DC	Degree Centrality
SC	Semi-local Centrality
PR	PageRank
CC	Closeness Centrality
SR	Similarity Ratio
BC	Betweenness Centrality
DR	Degree Ratio
TV	Trust value

## Appendix A

### Appendix A.1. Cumulative Infected Nodes for Proposed Centrality with Basic Centralities

We present the cumulative infected nodes or the effect of spreading the information while infecting the top-10 seed nodes or influential nodes in this part. The top-10 seed nodes were computed by the proposed centrality approach (NTPR); basic centrality measures BC, CC, SC, DC, PR, and TPR; and a greedy method. These top-10 seed nodes were initially infected via the SIR model. The neighboring vertices of these seed nodes were infected with infection probability β in the next time step. The infection probability β was estimated to be between 0.1 and 0.4. The top-ten influential nodes were discovered by NTPR, DC, CC, PR, BC, SC, TPR, and the greedy method using the SIR model for 4 different networks. For better understanding and analysis, we show Figure A1, Figure A2, Figure A3, Figure A4 and Figure A5.

The NTPR centrality produced a better outcome than PR, TPR, CC, DC, BC, SC, TPR, and the greedy approach in powergrid and euroroad networks. For email-univ and web-polblogs networks, our method performed similarly to the existing centralities.

### Appendix A.2. Maximum Influence at Various Infection Probabilities with Various Centrality Methods

We have shown the normalized maximum infection with different infection probability in Figure A6. We compare NTPR with TPR, PR, BC, SC, DC, and CC centralities and a greedy approach. In this section, we show the email-univ and web-polblogs networks. In both networks, the results of greedy, TPR, SC, CC, BC, and DC centralities all one. The PR had low performance for these networks. We display the average information spread with various infection probabilities in Figure A7. In the four datasets, the greedy method produced greater average information spread compared with other centralities.
